# Androgens Exert a Cysticidal Effect upon *Taenia crassiceps* by Disrupting Flame Cell Morphology and Function

**DOI:** 10.1371/journal.pone.0127928

**Published:** 2015-06-15

**Authors:** Javier R. Ambrosio, Laura Valverde-Islas, Karen E. Nava-Castro, M. Isabel Palacios- Arreola, Pedro Ostoa-Saloma, Olivia Reynoso-Ducoing, Galileo Escobedo, Azucena Ruíz-Rosado, Lenin Dominguez-Ramírez, Jorge Morales-Montor

**Affiliations:** 1 Departamento de Microbiología y Parasitología, Facultad de Medicina, Universidad Nacional Autónoma de México, D.F., México; 2 Departamento de Ciencias Ambientales, Centro de Ciencias de la Atmósfera, Universidad Nacional Autónoma de México, D.F., México; 3 Departamento de Inmunología, Instituto de Investigaciones Biomédicas, Universidad Nacional Autónoma de México, D.F., México; 4 Unidad de Medicina Experimental, Facultad de Medicina, Universidad Nacional Autónoma de México, Hospital General de México, D.F., México; 5 Division of Experimental Medicine, McGill University, Montreal, Quebec, Canada, Segar Cancer Center, Lady Davis Institute for Medical Research, Jewish General Hospital, Montreal, Quebec, Canada; 6 Departmento de Ciencias Químico-Biológicas, Escuela de Ciencias, Universidad de las Américas Puebla, Santa Catarina Mártir Cholula, Puebla, Mexico; Baylor College of Medicine, UNITED STATES

## Abstract

The effects of testosterone (T_4_) and dihydrotestosterone (DHT) on the survival of the helminth cestode parasite *Taenia crassiceps*, as well as their effects on actin, tubulin and myosin expression and their assembly into the excretory system of flame cells are described in this paper. *In vitro* evaluations on parasite viability, flow cytometry, confocal microscopy, video-microscopy of live flame cells, and docking experiments of androgens interacting with actin, tubulin, and myosin were conducted. Our results show that T_4_ and DHT reduce *T*. *crassiceps* viability in a dose- and time-dependent fashion, reaching 90% of mortality at the highest dose used (40 ng/ml) and time exposed (10 days) in culture. Androgen treatment does not induce differences in the specific expression pattern of actin, tubulin, and myosin isoforms as compared with control parasites. Confocal microscopy demonstrated a strong disruption of the parasite tegument, with reduced assembly, shape, and motion of flame cells. Docking experiments show that androgens are capable of affecting parasite survival and flame cell morphology by directly interacting with actin, tubulin and myosin without altering their protein expression pattern. We show that both T_4_ and DHT are able to bind actin, tubulin, and myosin affecting their assembly and causing parasite intoxication due to impairment of flame cell function. Live flame cell video microscopy showing a reduced motion as well changes in the shape of flame cells are also shown. In summary, T_4_ and DHT directly act on *T*. *crassiceps* cysticerci through altering parasite survival as well as the assembly and function of flame cells.

## Introduction

Sex hormones affect the course of helminth infection [1, 2], particularly in the case of the cestodes *Taenia crassiceps* and *Taenia solium* [3, 4]. It has been suggested that *T*. *crassiceps* can exploit the hormonal microenvironment within the host by a system of trans-regulation, in which the parasite exploits host hormones and growth factors to facilitate infection and potentially increase growth and reproduction rates [5]. Furthermore, endocrine factors, such as sex or age, are well recognized to be involved in the nature of the immune response to parasites [6] or in the direct effect on them [5]. Thus, steroid hormones play key roles in the susceptibility to murine cysticercosis at two levels: a) regulating the immune response [7–9] or b) by having a direct effect on the parasite development [7–11]. Steroid hormone effects are not only restricted to cestode parasites but also extended to nematodes such as *Ancylostoma caninum*, whose number of larval and adult stages is increased by sex steroid hormones in several organs of the mouse [12]. In the nematode human parasite *Trichinella spiralis*, it has been shown that progesterone and estradiol decrease the moulting rate of the larvae, though at a different rate than controls untreated. The effect of the aforementioned steroids was shown to occur in a concentration- and time-independent fashion [13]. Furthermore, it has been found that *in vitro* treatment with progesterone increases *T*. *solium* scolex evagination and worm growth in a concentration-independent fashion [10].

Murine intraperitoneal cysticercosis is caused by the taeniid *Taenia crassiceps*, which has been useful for exploring some physiological host factors associated with porcine cysticercosis, and to some degree, with human neurocysticercosis [10, 14, 15]. Murine intraperitoneal *T*. *crassiceps* cysticercosis [16, 17] is a convenient strategy that allows us to control and systematically reproduce experiments by generating numerical data of parasite loads in individual mouse in a matter of weeks after infection. Its general representation of other forms of cysticercosis has been strengthened by similar results in other mouse and parasite strains [17] by the parasite’s extensive sharing of antigens with other taeniids and cestodes [18] and by the DNA homology between *T*. *crassiceps and T*. *solium* [18, 19]. In *T*. *crassiceps* cysticercosis, the effects of testosterone (T_4_) or dihydrotestosterone (DHT) have been also demonstrated [7]. When gonadectomized males received androgen replacement therapy and gonadectomized female mice were administered with either T_4_ or DHT before infection, parasite loads decreased by 80% in both mice groups [7]. This effect was shown to be mediated through the immune system since T_4_ or DHT treatment induced significant recovery of the splenocyte proliferation and Th_1_ cytokine production in these animals, both of which are related to protection against the parasite [7].

Thus, notwithstanding the host immune response that deals with the parasite, the possibility of additional direct effects of steroid hormones upon the parasites’ physiology should not be discounted. For example, *Ancylostoma caninum* grows better and increases egg production when the host is injected with testosterone (T_4_) [12]; T_4_ increases viability of *Nematospiroides dubius* larvae in the gut of the rat [20], as it does with *Nippostrongylus brasiliensis*, located in the hamster gut [21]; *Leishmania major* systemic infections in mice are strongly affected by T_4_ [22] and larval development of intestinal cestodes are accelerated by T_4_ (*Echinococcus granulosus* [23] and *Mesocestoides corti* [24]). Furthermore, *in vitro* testosterone or dihydrotestosterone treatment of *T*. *crassiceps* decreases parasite proliferation, reproduction and survival [11], suggesting that androgens have toxic effects on the parasite.

Flame cells morphology in cestodes is formed of actin, tubulin, and myosin, that ensemble together to form a protein complex supporting the cells unique shape [25, 26]. The flame cells constitute the basic unit of the protonephridial system (PS) of invertebrates. In cestodes, the PS is the excretory system in charge of allowing parasites to conserve water and eliminate salts in order to survive in the intestine or body cavities of their hosts, where they act as osmoconformers [25, 26]. It has been previously shown that direct exposure of *T*. *crassiceps* cysticerci to estradiol (E_2_) and progesterone (P_4_) increases protein expression of actin, tubulin, and myosin. In association with this, there was an increase in flame cells assembly and function, which resulted in better growth of the parasite [27].

However, despite the fact that androgens have been shown to have cysticidal activities, the knowledge of the exact effects of androgens on the FC morphology and their role in the parasite physiology is still lacking, and there is no clear explanation of the molecular mechanisms mediating such cysticidal function.

Our study was then designed to thoroughly explore the *in vitro* effects of testosterone and dihydrotestosterone upon the metacestode stage of *T*. *crassiceps*. We demonstrated by ultrastructural morphology, immunofluorescence, immunochemistry and flow cytometry analysis, that T_4_ and DHT do not affect the distribution and expression of actin, myosin and tubulin isotypes in tegumental tissues. However, using confocal microscopy, we demonstrated that flame cell morphology and tegumental integrity were affected. By using live flame cell video microscopy, we demonstrated that *in vitro* androgen treatment affects the motion and shape of flame cells, thereby inhibiting their detoxification activities and increasing parasite mortality as the result of the accumulation of toxic products of its metabolism. Furthermore, docking experiments demonstrated that both T_4_ and DHT are able to bind to actin, tubulin and myosin, interfering with flame cell assembly in an allosteric manner. To our knowledge, this is the first report to demonstrate the effects of T_4_ and DHT on flame cells and may potentially benefit not only the understanding of the host-parasite molecular cross-talk, but also the design of drugs that specifically arrest the activity of important parasite molecules such as proteins involved in detoxification, thus affecting their establishment, growth and reproduction in an immunocompetent host.

## Materials and Methods

### Ethics Statement

Animal care and experimentation practices at the Instituto de Investigaciones Biomédicas are constantly evaluated by the Institute´s Animal Care and Use Committee, adhering to the official Mexican regulations (NOM-062-ZOO-1999). Mexican regulations are in strict accordance with the recommendations in the Guide for the Care and Use of Laboratory Animals of the National Institute of Health (NIH) of the USA to ensure compliance with established international regulations and guidelines. To obtain parasites, mice were sacrificed using sodium pentobarbital anaesthesia to obtain parasites. Efforts were always made to minimize suffering.

Female Balb/c AnN (H2-d) inbred mice obtained from Harlan (Mexico City) were used in all of the experiments. Animals were housed in the animal care facilities at the Instituto de Investigaciones Biomédicas (UNAM), under controlled conditions of temperature (22°C) and 12 h dark-light cycles with lights on between 0700 and 1900. They were fed Purina Diet 5015 (Purina, St. Louis, MO) and tap water ad libitum. The fast-growing ORF strain of *T*. *crassiceps* was used for infection. Ten non-budding *T*. *crassiceps* larvae (approximately 2 mm in diameter) were suspended in 0.3 ml sterile phosphate-buffered saline (PBS: 0.15M NaCl, 0.01M sodium phosphate buffer, pH 7.2) and carefully injected intraperitoneally into 42day-old female mice using a 0.25 gauge needle. Infected mice were housed in separated cages (5 each) in the same room of the animal facility. At 16 weeks of infection, mice were rapidly euthanized by cervical dislocation after anesthesia with pentobarbital (Pfizer, Mexico), always at 08:00 AM.

### Parasites

Cysticerci were obtained from intraperitoneally infected mice and placed in tubes containing sterile PBS (1x) supplemented with 100 U/ml of the anti-infective Fungizone (Gibco, Grand Island). The tubes were centrifuged for 10 min at 1,500 rpm and 4°C, and the supernatant were discarded. Packed cysticerci were incubated in DMEM serum-free medium (Gibco 12491). They were then washed by centrifugation three times for 10 min at 1500 rpm. After the final wash, the numbers of viable cysticerci (complete, translucent and motile cystic structures) were counted under a binocular microscope. Ten viable non-budding cysticerci of approximately 2-mm diameter were then selected and dispensed into each well of 24-well culture plates (Falcon, Becton Dickinson Labware, Franklin Lakes, New Jersey) in 1 ml DMEM Medium (Gibco 12491) and incubated at 37°C and 5% CO_2_. A sufficient number of culture wells were prepared to accommodate the complete experimental design to evaluate the effects of *in vitro* treatment of testosterone and dihydrotestosterone on cysticerci. Cultures were checked daily and their medium was completely replaced every other day.

### *In vitro* treatment effects of T_4_ and DHT on *T*. *crassiceps* cysticerci reproduction

Culture grade testosterone (T_4_) and dihydrotestosterone (DHT) were obtained from Sigma. For *in vitro* tests, T_4_ and DHT were dissolved in pure ethanol (Sigma) to the desired stock concentration and sterilized by passage through a 0.2 mm Millipore filter. Afterwards, hormones diluted in ethanol were left to evaporate by heating at 37°C, and then resuspended in culture medium. The experiments used the parasite-loaded wells: six wells were used as untreated controls, six wells were treated with different concentrations of T_4_ (0, 0.5, 1, 2, 4, 8 and 16 nM) and six wells with increasing concentrations of DHT (0,0.5, 1, 2, 4, 8 and 16 nM). Each hormone was prepared in a final volume of 100 μl and added to 2 ml of medium in each well. A total of six different experiments were performed. In hormone dose-response curves, only the motility and viability in ten days in culture were assessed as the response variables. From the dose-response curves of each hormone, an optimal dose was selected to use in further experimentation: the dose of each hormone at the shortest time at which the differences from the respective control values were maximal. Thus, for T_4_ it was 8 nM and for DHT 4 nM. Visual assessment of motility and viability was determined daily for the various wells using an inverted microscope (Olympus, MO21, Tokyo, Japan) at 10x and 100x magnification. Injury to cysticerci was recognized microscopically by progressive internal disorganization, development of white opaque areas in the parasites’ tegument and by loss of their motility. Dead cysticerci were immobile, opaque and disorganized structures.

### Specific detection of actin, tubulin and myosin in *T*. *crassiceps* cysticerci by flow cytometry

Flow cytometry experiments were conducted as previously described [27, 28]. Briefly, *T*. *crassiceps* cells were extracted by tissue disruption from cultured control, T4 or DHT treated parasites. Mouse spleen cells were used as FACS calibration controls. For each treatment, 2 x 10^6^ cells were suspended in 100 μl of fixation buffer (PBS, 2% para- formaldehyde) and incubated at 37°C for 10 min. The cells were then permeabilized in 1 ml of frozen methanol at 4°C for 30 min. Next, the cells were centrifuged at 2200g for 5 min and washed three times with staining buffer (PBS, 2% FBS, 0.02% sodium azide).

The cells were incubated with anti-actin clone C4 (1:100, Millipore, California, USA), anti-α-Tubulin clone DM-1A (1:100, Sigma), or rabbit polyclonal anti-taeniid myosin II as described previously [29] (1:50) at room temperature (RT) for 20 min and washed in staining buffer. Paramyosin antibody, produced in our laboratory, was only used to discriminate among *T*. *crassiceps* and host cells.

Then, the cells were pelleted at 2200g for 5 min and resuspended separately with FITC-conjugated goat anti-rabbit or PE-conjugated rat anti-mouse for 30 min at 4°C in the dark. Next, the cells were washed in staining buffer and centrifuged at 2000 rpm for 5 min; the pellets were resuspended in 500 μl of staining buffer in the dark and analysed by flow cytometry on a FACS Calibur (BD Biosciences). The data were analysed with FlowJo version 8.7.

### Laser Scanning Confocal Microscopy (LSCM)

Parasites were treated and processed for fluorescence microscopy as previously reported [26]. Briefly, parasites were embedded in Tissue-Tek O.C.T. (Sakura Finetek) and frozen in liquid nitrogen, and 10-μm thick sections were prepared in a cryostat (CM Leica 1100) and fixed in cold acetone. The distribution of cytoskeletal proteins was analysed as follows: for actin, cryosections were incubated with Rhodamine-coupled phalloidin (Invitrogen) diluted to 1:40 for 1 h at room temperature (RT). For myosin II, cryosections were incubated for 1 h at RT with a polyclonal primary antibody raised against *T*. *solium* myosin II [30] diluted to 1:1000 and followed with biotinylated anti-rabbit IgG (H+L) antibody (Vector Lab. Burlingame) diluted to 1:30 for 1 h and subsequently with the avidin-rhodamine complex diluted to 1:50 for 1 h in the dark. For α-tubulin, cryosections were incubated with a commercial DM1A antibody (Santa Cruz Biotechnology, Inc.) diluted to 1:100 for 1 h at RT, and anti-mouse IgG conjugated to FITC was added at 1:30 dilution. Nuclei were stained with DAPI (50 μg/mL) (Sigma) for 30 min at RT. All preparations were washed with PBS and later embedded on a mounting medium (DAKO). Controls were prepared as described above, with the omission of primary antibody. Observations were performed in an Olympus FluoView FV1000 confocal microscope using the objectives 10X (UPLSAPO), 40X (UPLFLN) and 100X (UPLSAPO). Nuclear DNA staining was performed by adding Propidium Iodide (Sigma) (1:1000) or DAPI (Sigma) (1 mg/ml) 5 min before the slides were examined. For observation, slides were washed with PBS and mounted in a commercial mounting solution for preserving fluorescence (DAKO). Control observations were conducted on parasite cryosections only incubated with secondary antibodies. Unless described, all reagents were from Sigma.

### Docking of testosterone and dihydrotestosterone to parasites’ tubulin, actin and myosin

Initial model generation was accomplished by using the sequences for actin, myosin VIb and tubulin-1-α and submitting them to Rosetta Homology modelling [31]. Resulting models clustered close together for the selection of the best model. Then they were submitted to energy minimization using Amber12 [32] and their quality was evaluated using Molprobity [33]. The highest quality model was selected to perform ligand docking. Blind docking was performed using Vina 1.1.2 on a 12-core computer running Mac OS X. All ligands were obtained from the ZINC database and converted to PDBQT format using the GUI provided by Autodock Tools. Ligands were checked manually against the known chemical structure using ChemAxon’s Marvin; all of their rotatable bonds were allowed to remain free from restraint during docking. The receptor (actin, myosin or tubulin-1-α, respectively) was kept rigid. Docking employed a grid of dimensions 40 x 40 x 40 with a 1 Å grid size. Exhaustiveness was always set to 5000. Analysis of the docking results was performed in PyMOL (DeLanoScientific, 2009), Daniel Seelinger’s Autodock/Vina plugin and the NNscore2 neural-network docking scoring protocol [34]. The results presented are the best candidates selected from the consensus score. The best results of the docking of DHT or testosterone to actin coincide with the interface between actin and myosin, according to electron microscopy data from insect flight muscle [35]. Docking of DHT or testosterone to tubulin-1-α results show both ligands at a site near the putative GTP binding site. It is unclear if DHT or testosterone binding could prevent GTP binding or just lower the affinity of the latter. Results for myosin VIB docking are difficult to interpret because the model is different from other myosins of known structures. As in the case of actin and tubulin-1-α, however, DHT and testosterone bind to the same site.

### Live-Cell Video microscopy

Live cysticerci were maintained for one or two days *in vitro* in RPMI 1640 medium supplemented with 25 mM HEPES buffer adjusted to pH 7.2 and 30 mM carbonate salts. Parasites were maintained in a humidified incubator at 37°C in a 5% CO_2_ environment.

For filming motion of FC in live parasites, cysticerci were punctured with a needle to eliminate the vesicular fluid and observations were performed on the internal side of the bladder walls, by spreading the tissue on a microscope slide and observing it directly using Nomarsky differential-interference-contrast microscopy (DIC). Time-lapse recordings of FC dynamics, at room temperature (RT), were carried out as follows: Each frame was captured at the rate of 3.2 s/frame producing a total of 34 frames using a confocal microscopy (Olympus FV1000) using a 60x objective, PLAPON 1.5 NA coupled to the software FluoView Ver. 2.1c.

### Experimental design and statistical analysis

T_4_ and DHT concentration-response and time-response curves were estimated in six independent experiments, each performed with ten cysticerci, freshly extracted from different infected donor female mice, each one replicated in 24 different wells. The response variable used in statistical analysis was the viability in the 24 wells with each treatment, along with the time of exposure of each experiment. Data of the six replications of each experiment were pooled and expressed as their average ± standard deviation. The mean of the fluorescence in the flow cytometry analysis was calculated for four different experiments and expressed as the average ± standard deviation. Data were analysed using one-way ANOVA and a subsequent Dunnet’s Multiple Comparison Test. Differences were considered statistically significant when P<0.05.

## Results

### *In vitro* viability of T_4_ and DHT-treated cysticerci

Table 1 shows the numerical results obtained in these experiments and the results of ANOVA testing, which detects the significant contribution to variance of the hormones employed, their doses and the time of exposure as single factors and with significant interactions between the factors. Different concentrations of T_4_ and DHT were studied *in vitro* to ascertain a dose-dependent response pattern upon *T*. *crassiceps*. There was a dose-response pattern when androgens were tested: T_4_ at a dose of 2 nM reduced parasite viability by 75% while a decrease to 50% was observed at 4 nM. DHT showed a somewhat stronger inhibitory effect than T_4_: a dose of 1 nM reduced viability by 85% whereas a 70% decreasing was seen at 2 nM. At 8 nM T_4_ reduced parasite viability to only 10%, and all cysticerci were dead using 16 nM. On the contrary, DHT-treated cysticerci were dead at 8 nM. All of the T_4_ and DHT-treated cysticerci were significantly smaller (218 ± 7.4 microns) (*P<0*.*05*.) than control cysticerci (1524 ± 94.9 microns), exhibiting progressive internal disorganization and development of opaque white masses in their inner milleu, as well as progressive loss of motility.

**Table 1:** Dose-response curves of testosterone (T_4_) and dihydrotestosterone (DHT) effects on the motility and viability of cysticerci of *Taenia crassiceps* ORF strain.

*T*_*4*_ *dose (nM)*	*Viability (%)*	*DHT dose (nM)*	*Viability (%)*
**0**	100 ± 0	**0**	100 ± 0
**0.5**	100 ± 0	**0.5**	100 ± 0
**1.0**	100 ± 0	**1.0**	85 ± 0
**2.0**	75 ± 12†	**2.0**	70 ± 0†
**4.0**	50 ± 7†	**4.0**	40 ± 11†
**8**	10 ± 0†	**8**	0 ± 1†
**16**	0 ± 0†	**16**	0 ± 0†

Data represents mean ± SD from 6 experiments with six wells by dose, and 10 cisticerci by well. The media and hormones were changed every other day, and cysticerci were in culture a total of 10 days.

^†^ *P< 0*.*05*,

^††^ *P< 0*.*01* of statistical significance.

### Actin, Tubulin and Myosin protein expression in *Taenia crassiceps* control, T_4_ and DHT-treated cysticerci by flow cytometry

**Fig 1:**
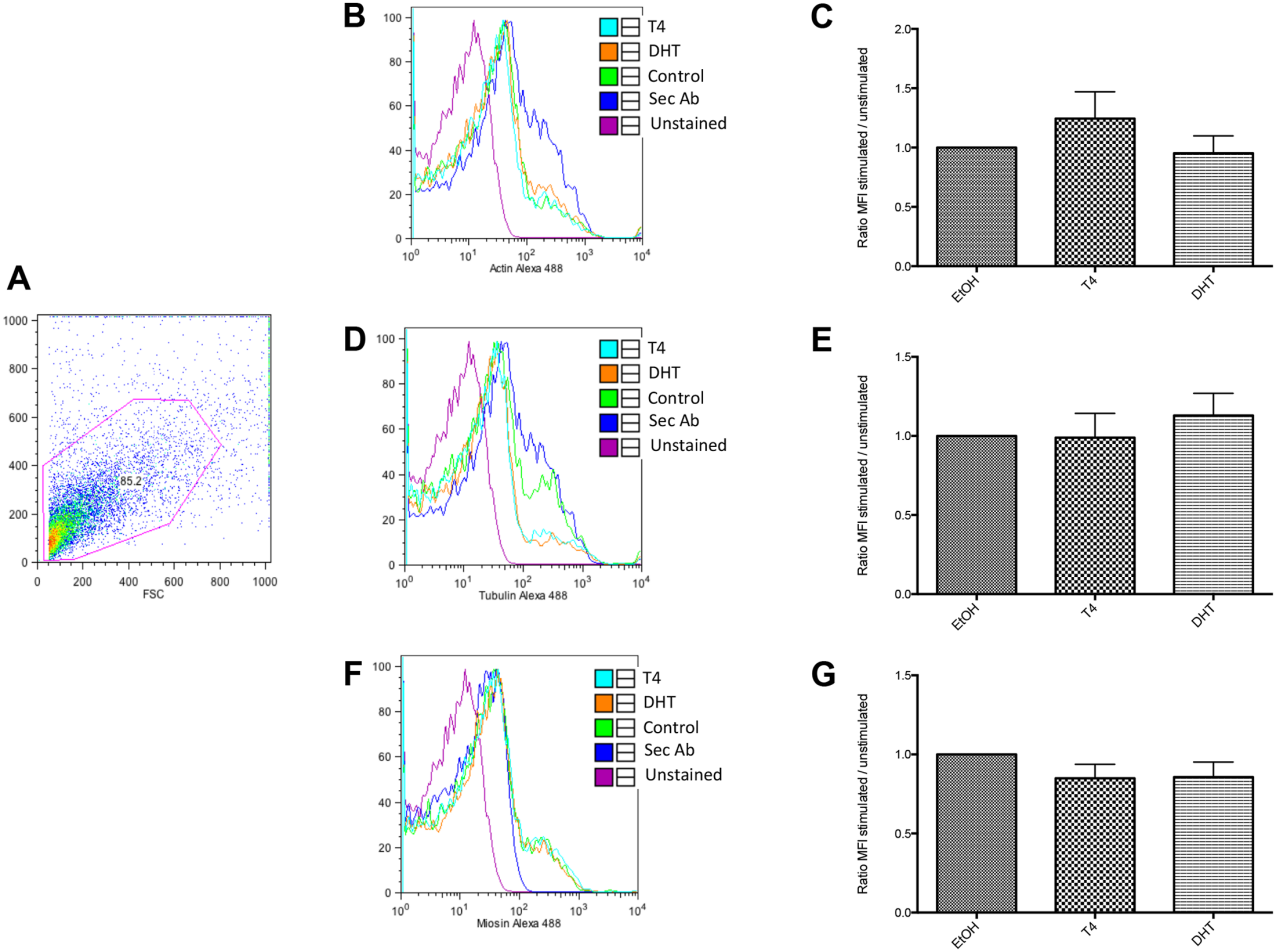
Specific expression of actin, tubulin and myosin in *Taenia crassiceps* cysticerci by flow cytometry. Dot plot depicted in Fig 1 (A), represents size and complexity of the *T*. *crassiceps* cells used for staining and detection of cytoskeletal proteins. FACS analysis of parasitic actin (B and C), tubulin (D and E) and myosin (F and G). Histograms (B, D and F) show a representative experiment of actin, tubulin and myosin-detection. Pink line: Unstained cells cultured in the presence of vehicle; Purple line: Unspecific secondary staining from FITC- (Sec Ab); Green line: Actin (B), Tubulin (D) or Myosin (F) specific expression in unstimulated cells cultured in the presence of vehicle; Light blue: Actin (A), Tubulin (C) or Myosin (E) specific expression in T4-stimulated cells; and Orange line: Actin (A), Tubulin (C) or Myosin (E) specific expression in DHT-stimulated cells. Panels (C, E and G) show the relative expression in steroid-treated and control cells. Relative expression was calculated according to: MFI of actin, tubulin or myosin stained cells / MFI secondary antibody stained cells. Data show the mean ± SE of five independent experiments.

In Fig 1A, it is shown the size and complexity of parasite-derived cells, used for flow cytometry. Histograms in Fig 1 show the expression of actin (B), tubulin (D), and myosin (F) in control (EtOH-cultured, green line), T_4_- (light blue) or DHT-stimulated cells (orange line). Pink and purple lines represent unstained cells and the unspecific staining of secondary antibodies (Sec Ab) (respectively). Bar graphs show the quantification of the relative expression of actin (C), tubulin (E) and myosin (G) in control (EtOH-cultured), T_4_- or DHT-cultured cells. Relative expression was calculated by dividing the mean fluorescence value from stained cells (in control, T_4_- or DHT-stimulated cells) by the mean fluorescence value of the unspecific staining of the secondary antibody-stained cells. Flow cytometry analysis showed that neither actin, or tubulin nor myosin changed among different treatments.

### Confocal microscopy of flame cells from control, T_4_ and DHT-treated cysticerci

**Fig 2:**
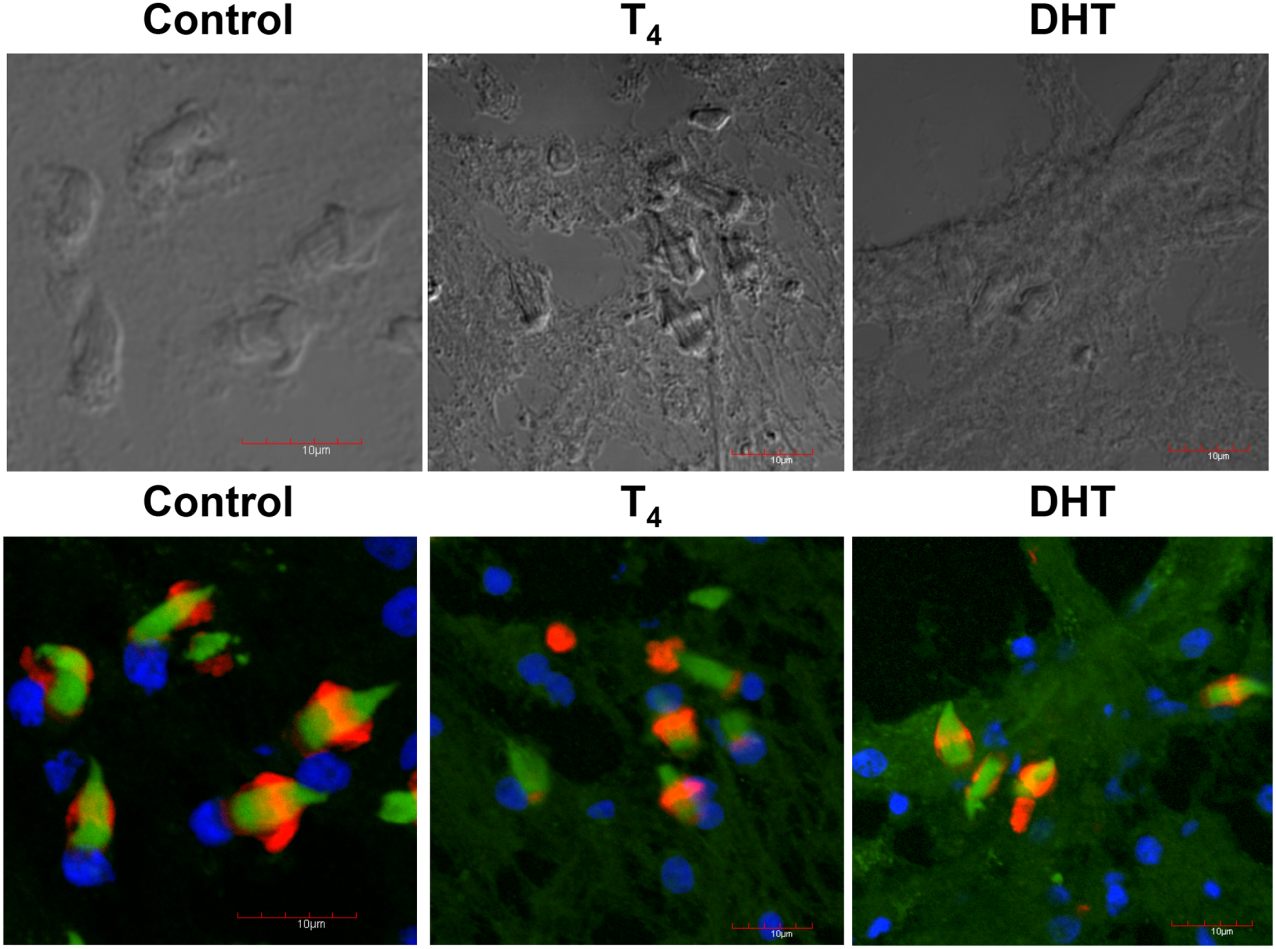
Confocal microscopy depicting flame cells of control, T_4_ and DHT-treated parasites. Phase contrast of the control sections of the slides obtained by Tissue-Teck fixing are shown in upper panel from left to right, control (C), testosterone (T_4_) and dihydrotestosterone (DHT). In lower panel, flame cells of control, cysticerci treated with T_4_ and DHT are presented. The normal number and distribution of flame cells is observed in control, while in those parasites exposed to T_4_ and those exposed to DHT there is a decreased and disrupted pattern of flame cells. Scale bar corresponds to 10 μm.

In the upper panel, it is shown the phase contrast of the samples used in control (EtOH), T_4_ and DHT treated cysticerci. No staining was significantly detected using only the secondary antibody for the detection of α-tubulin and myosin (not shown). Normal, well-developed flame cells of control parasites are presented in the lower panel. Also, in this panel, it is seen the normal number and distribution of flame cells (Fig 2, lower panel). However, flame cells (FC) of cysticerci treated with T_4_ and DHT did lose cytoskeletal normal distribution of the associated nuclei and proteins (actin in green, tubulin in red and DAPI in blue) also showing a decrease in the number and assembly of cytoskeletal proteins in comparison with similar FC of parasites from the control group (Fig 2, lower panel). Also, it is worthy of mentioning that there was an extremely high level of disorganisation in the parasite’s tegument in both T_4_ and DHT-treated cysticerci. Additionally, DHT treatment induced an apparent decrease in the expression of α-tubulin, F-actin, and nuclei after their staining with their respective fluorescence marker as indicated. The tegument seemed to be teared up and some holes were clearly shown (Fig 2, lower panel).

### Confocal microscopy of nuclei from control, T_4_ and DHT-treated cysticerci

**Fig 3:**
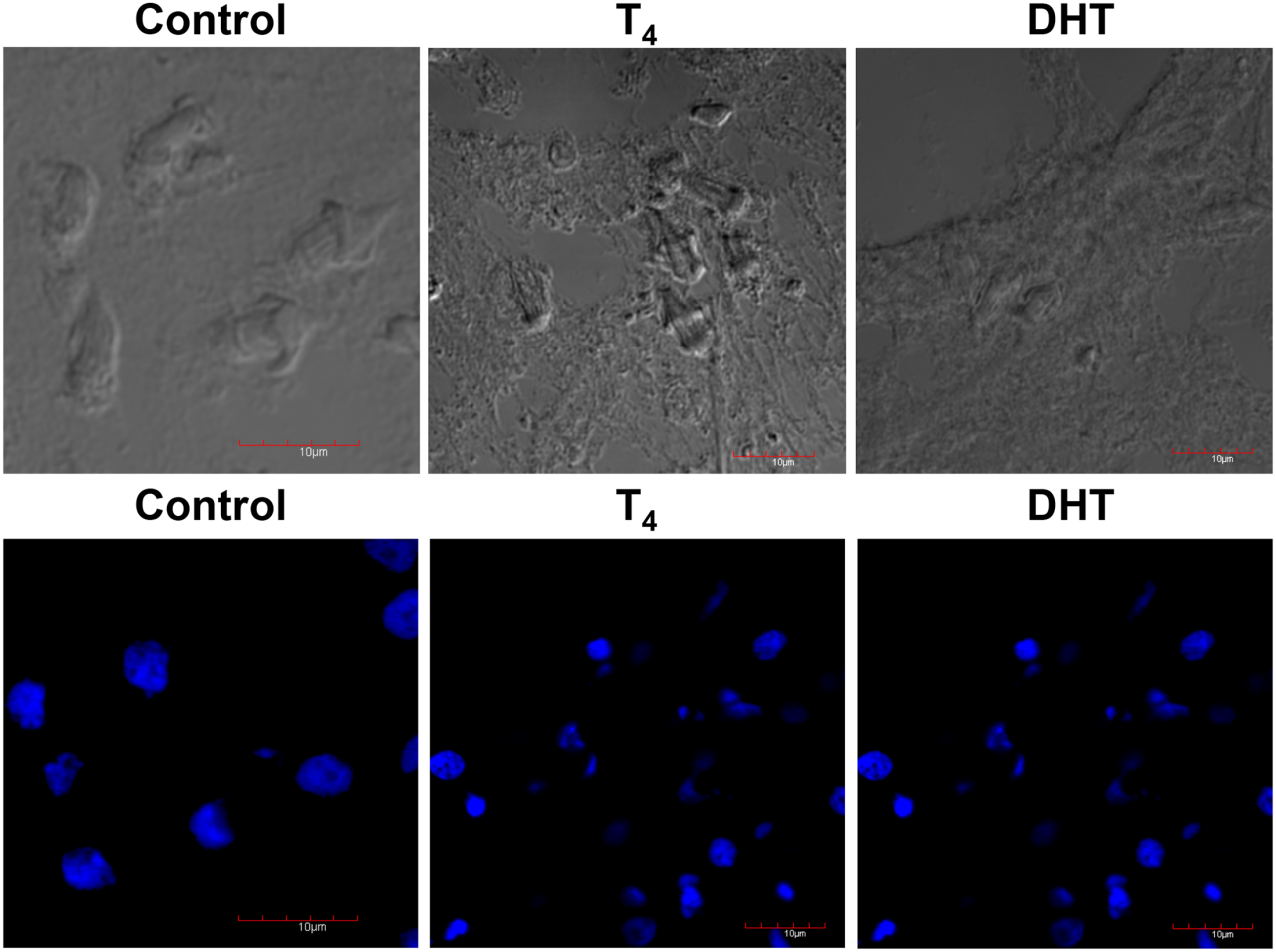
Immunolocalization of nuclei after staining with DAPI of control, T_4_- and DHT-treated cysticerci. Phase contrast of the control sections of the slides obtained by Tissue-Teck fixing are shown in upper panel from left to right, control (C), testosterone (T_4_) and dihydrotestosterone (DHT). In lower panel, DAPI was used to detect nuclei in frozen tissue sections fixed with Tissue-Teck. Treatment with both steroids induced changes in the distribution of nuclei, which also affected the morphology of FCs. Cysticerci were observed under an SEM Zeiss DSM-950 after five days of treatment with 50 μg/ml of each steroid. Scale bar corresponds to 10 μm.

In Fig 3, once again, in upper panel it is shown the phase contrast of the samples used in all treatments. Due to the treatment with T_4_ and DHT (lower panel), fewer tiny blue spots were associated with the parasite’s tegument. Higher magnifications of the tiny blue spots showed that they correspond to the distribution of nuclei of FC (lower panel). Such a marked reduction in the nuclei contents seems to be in agreement to previous reports showing that testosterone and DHT have anti-reproductive effects upon *T*. *crassiceps* (Fig 3, lower panel).

### Confocal microscopy and immunolocalization of α-Tubulin from control, T_4_ and DHT-treated cysticerci

**Fig 4:**
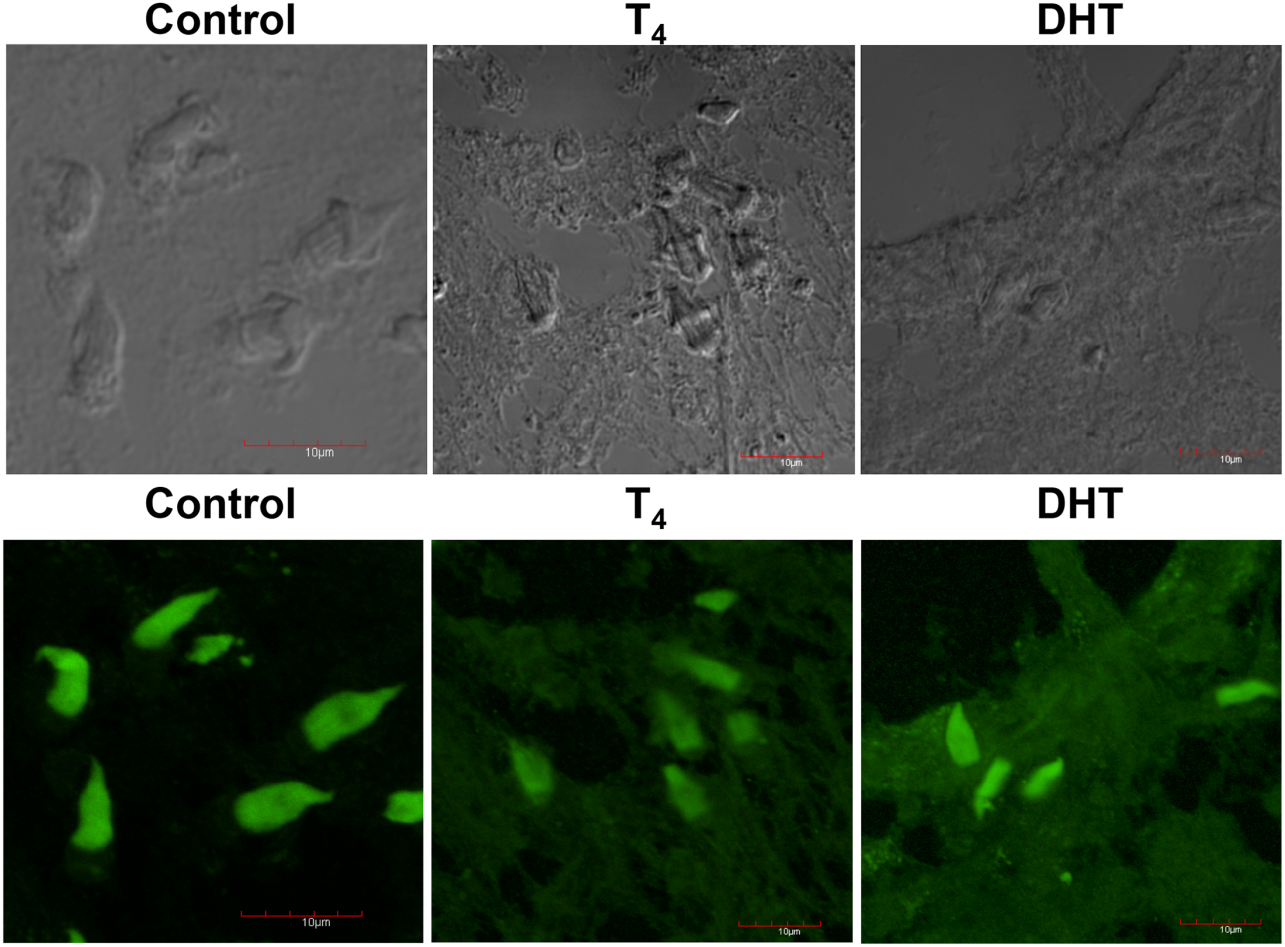
Immunolocalization of α-tubulin (green) of control, T_4_ and DHT treated parasites. Phase contrast of the control sections of the slides obtained by Tissue-Teck fixing are shown in upper panel from left to right, control (C), testosterone (T_4_) and dihydrotestosterone (DHT). In lower panel, green tiny spots are produced by the specific binding of the anti-α-tubulin stained with alexa-488 antibody to cytoskeletal α-tubulin. Tubulin protein inside the bladder wall and at the level of the tegument in the tissue of control parasites and those treated with T_4_ and DHT. Scale bar corresponds to 10 μm.

In the upper panel, it is shown the phase contrast of the samples used in all treatments. No staining was significantly detected using only the secondary antibody (not shown). In lower panel, green-fluorescent α-tubulin was associated to flame cell ciliary tufts, as is shown in control untreated cysticerci (Fig 4). As previously indicated, alterations in the distribution of cytoskeletal proteins in FC were induced because of the treatment with T_4_ (Fig 4, lower panel). A similar distribution of cytoskeletal α-tubulin in the FC of parasites treated with DHT is shown in Fig 4, lower panel.

### Confocal microscopy and immunolocalization of F-actin from control, T_4_ and DHT-treated cysticerci

**Fig 5:**
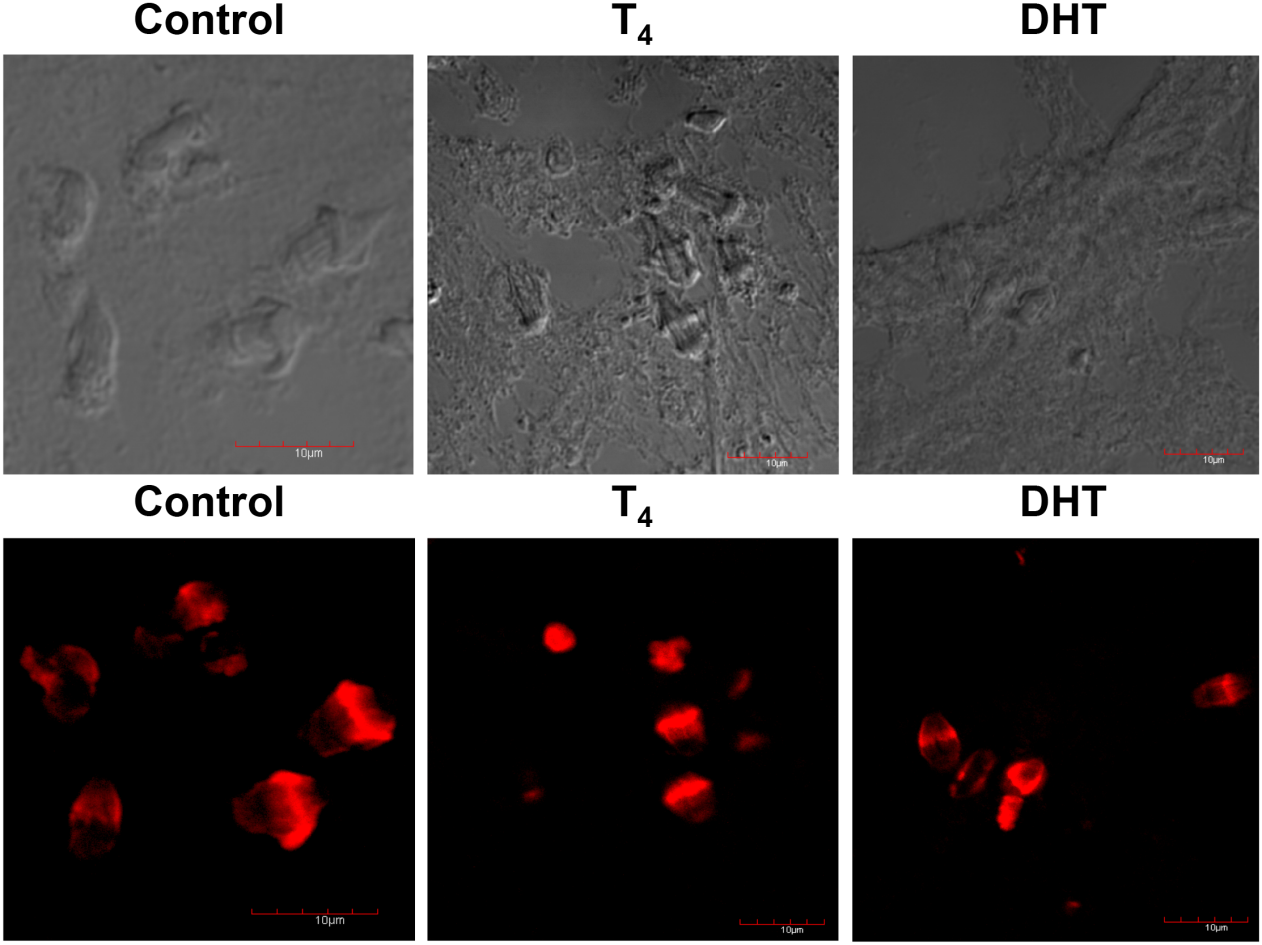
Immunolocalization of F-actin (red) of control, T_4_ and DHT treated parasites. Phase contrast of the control sections of the slides obtained by Tissue-Teck fixing are shown in upper panel from left to right, control (C), testosterone (T_4_) and dihydrotestosterone (DHT). In lower panel, red tiny spots are produced by the specific binding of the anti-α-tubulin stained with rhodamine-coupled phalloidin to cytoskeletal F-actin. F-actin proteins inside the bladder wall and at the level of the tegument in the tissue of control parasites and those treated with T_4_ and DHT. Scale bar corresponds to 10 μm.

Cysticerci of the control group presented a specific localization of F-actin in the parasite tegument (Fig 5, lower panel). Positive staining for F-actin is shown in red. Tiny red spots already demonstrated positive expression of F-actin (Fig 5). F-actin expression was apparently decreased because of the treatment with T_4_ (Fig 5, lower panel), while DHT exhibited the opposite pattern (Fig 5, lower panel).

### Docking of T_4_ and DHT to *T*. *crassiceps* actin, tubulin and myosin

**Fig 6:**
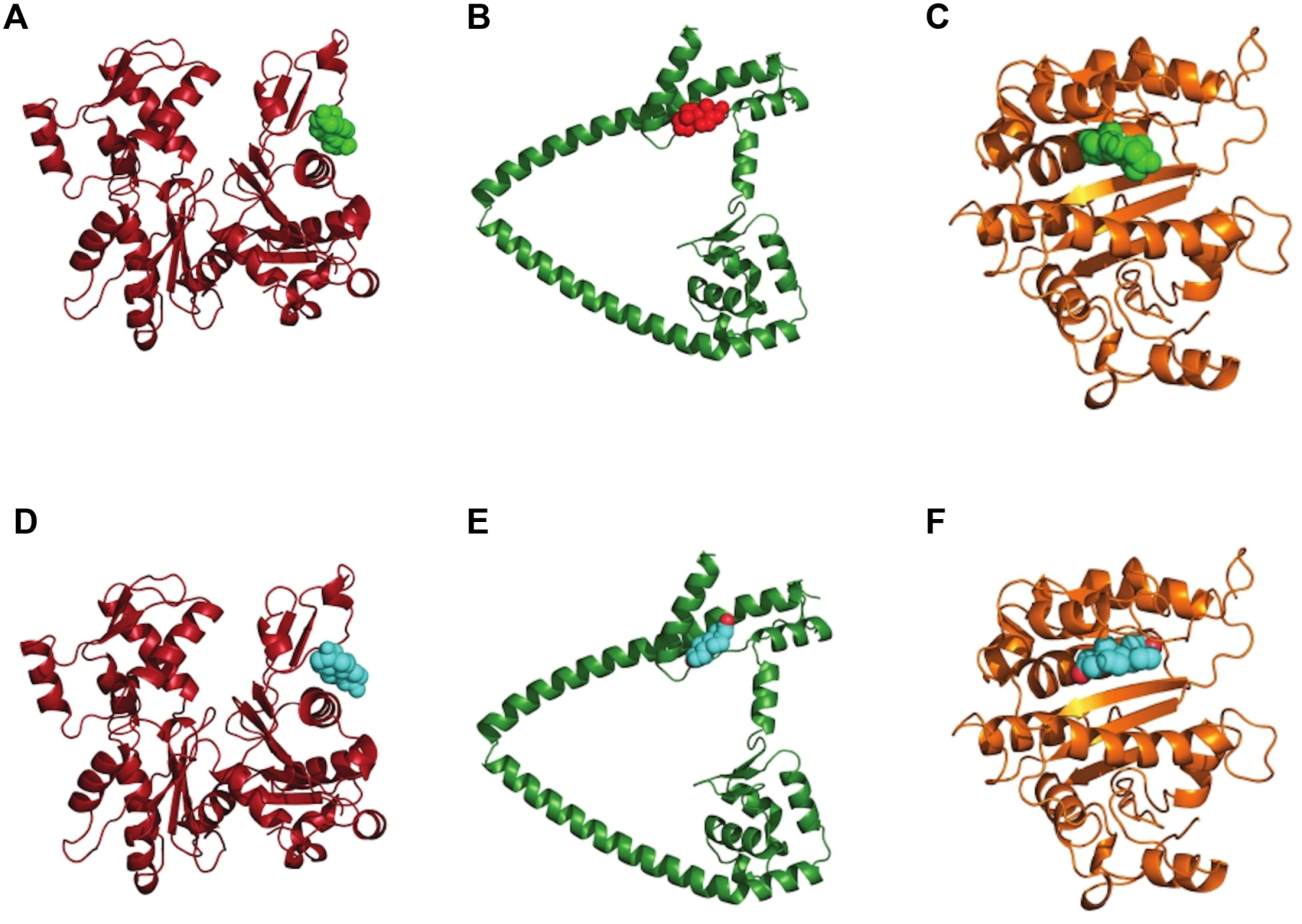
Docking of testosterone or dihydrotestosterone to actin, tubulin and myosin. Dihydrotestosterone (green or red) and testosterone (cyan) docking to actin (A and D), myosin VIb (B and E) and tubulin (C and F). We show the higher affinity sites according to neural-network scoring.

The best results for docking of DHT or testosterone to actin coincide with the interface between actin and myosin, as in the electron microscopy data from insect flight muscle [35]. Docking data of DHT or testosterone to tubulin-1-α show both ligands at a site close to the putative GTP binding site. It is unclear if DHT or testosterone coupling can prevent GTP binding or just decreases affinity of the latter one. Results for myosin VIB docking are difficult to interpret because the model is different from other myosin proteins of previously known structures. However, as in the case of actin and tubulin-1-α, DHT and testosterone seem to be capable of binding to the same site (Fig 6).

### Video microscopy of flame cells of control, vehicle and T_4_ and DHT-treated cysticerci

We used video to illustrate the motions of the cytoskeletal proteins of a *T*. *crassiceps* control FC (S1 Movie) and vehicle-treated FC. Through video microscopy, we are showing the potential ways of delivering substances to the cell body of the parasite, as well the interior of vesicles produced by invagination of the plasmatic membrane and secondly, the vesicular trafficking from extended processes of cells in communication. In S1 Movie, the movement of flame cells of control and vehicle treated parasites is observed, and those treated with T_4_ and DHT are observed in S1 and S3 Movies. It is clear that hormonal treatment alters both the normal flame cell morphology and movement speed, thus affecting secretion of toxic compounds inside the parasite. In this way, T_4_ and DHT do not only decrease the number of flame cells but also disrupt their function (S2 and S3 Movies).

## Discussion

Our study shows that androgens act directly upon *T*. *crassiceps* cysticerci viability without needing the host’s participation: T_4_ and DHT lead to the irreversible cellular damage of cysticerci. This effect depends on both concentration and duration of exposure to the mentioned hormones. The effects on reproduction and viability began after 24 h in culture, but were maximal between experimental and control groups at 5–10 days of culture. DHT has more drastically deleterious effects upon the cysticerci than T_4_ does. Previously, we had found that gender and circulating T_4_ and DHT levels in host mice crucially affect the dynamics of parasite loads in mice infected with *T*. *crassiceps* cysticerci [7, 36].

The effects of T_4_ and DHT can be directly associated with their physicochemical properties and their mode of entry into the parasite tissues. The chemical structure of the steroids may favour any effective interaction with the tegumental surface. We recently described an oestrogen receptor on the tegument of the parasite [37]. Thus, androgens can bind to a steroid hormone receptor and interact with the surface of parasites, as deep cells of the germinal zones do with flame cells. However, there is no description of an androgen receptor in *T*. *crassiceps* that can explain its effects to date. Perhaps passive diffusion through tegument is the explanation by which androgens easily reach the inner parasite tissues. It has been established that passive diffusion is the main mode of entry for some drugs with similar structure that androgens, with passage through the cuticle of nematodes and the tegument of cestodes and trematodes [38]. It is likely that the higher lipophilicity of androgens increases their ability to cross the tapeworm. This means that if androgens have a higher lipophilicity, they easily undergo a process of diffusion, internalization and accumulation in *T*. *crassiceps* cysticerci, favouring their arrival at the internal layers of the vesicular bladder wall and interacting directly with cells located at the germinal layer. Specific interactions of androgens with tubulin isotypes of microtubules of ciliary tufts apparently provoke instability of the microtubules of cilia and cause changes in flame cell morphology and an increase in the beating of the cilia (S2 and S3 Movies). It has been established that the dynamics of the microtubules are important for the exact organization of intracellular events that involve organelle movements and the maintenance of several protein networks, such as those produced by cytoskeletal proteins [39]. In the presence of T_4_ and DHT, it was clear that changes were produced in the shape, length, and structure of cilia of flame cells of *T*. *crassiceps* cysticerci. The importance and implications of these studies can be extended to the Cestoidea class and can also be applied to the *Platyhelminth* phylum. Better knowledge of the regulation of the expression of protein components and the composition and dynamics of flame cells could give a better understanding about the terminal cells of the complex excretory systems of these organisms and how products of the host affect their physiology, as may be the case of hormones [25]. In addition, for medically important helminths, this knowledge could contribute to better design of anti-helminthic drugs because microtubules are the main cytoskeletal proteins for an accurate function of flame cells and thus the parasite excretory system [40]. To survive inside the host, cysticerci need to carry out continuous turnover of substances, for which the maintenance of an intact excretory system appears to be crucial [41].

Flame cells cannot be seen by direct observation of live, intact *T*. *crassiceps* cysticerci because the parasite tissue layers (brush border, syncytial tegument, and myocyte fibres) impede their visualization. Punctured parasites were used to observe the flickering flame cells from the inner bladder wall, where they were easily identified by the typical movements of beating cilia (S1 and S2 Movies). The behaviour of the ciliary tufts in flame cells was found to be similar to that described previously for *T*. *solium* [42] and other cestodes [43] and further indicates that these ciliary cells are largely dynamic. Present findings corroborate and strengthen previous results that show a marked concentration- and time-dependent pattern in the effects of T_4_ and DHT on cysticercus reproduction and welfare. In addition, this finding offers an alternative explanation about why *T*. *crassiceps* cysticerci grow better in a low androgen environment as that provided by female mice [42] and why parasites do not grow well in a high androgen environment, emphasizing the molecular cross-talk between host and parasite, which is in turn differentially influenced by the hormonal microenvironment of each gender.

One intriguing question is how androgens affect parasite survival through flame cell function, without inducing changes in actin, tubulin, and myosin expression. The key factor could be in the molecular structure of androgens and the aforementioned proteins. Docking experiments of both actin and tubulin-1-α result in two interesting possibilities for blocking the normal action of those proteins. Firstly, interaction between actin and DHT could block the binding of myosin to actin filaments. It will result in different phenotypes depending on the target tissue. Secondly, in the case of tubulin-1-α, interaction between tubulin and DHT could prevent GTP binding or slow its hydrolysis affecting microtubule assembly.

## Conclusions

Our results succeed in contributing data about the mechanisms by which the host microenvironment affects the parasite. Furthermore, the evolutionary origin of the molecules described herein, which facilitates exploitation of the host’s hormones, is worthy of keeping on study. The fact that testosterone and DHT seem to be capable of interfering with the development of *T*. *crassiceps* cysticerci could find important application in the development of future anti-cestode drugs and therapeutic protocols for treating cysticercosis in humans and cattle.

## Supporting Information

S1 Movie Motion of cytoskeletal proteins in *T*. *crassiceps* control FC by video microscopy. Movement of FCs from media control parasites is shown.
(MOV)

S2 Movie Motion of cytoskeletal proteins in testosterone-treated *T*. *crassiceps* FCs by video microscopy. Movement of FCs from T_4_-treated parasites is shown.
(MOV)

S3 Movie Motion of cytoskeletal proteins in dihydrotestosterone-treated *T*. *crassiceps* FCs by video microscopy. Movement of FCs from DHT-treated parasites is shown.
(MOV)
